# Association between Pulmonary Function and Stair-Climbing Test Results after Lung Resection: A Pilot Study

**DOI:** 10.1155/2018/1925028

**Published:** 2018-09-09

**Authors:** Yohei Kubori, Ryosuke Matsuki, Akira Hotta, Tomoyuki Morisawa, Akira Tamaki

**Affiliations:** ^1^Department of Rehabilitation, Kansai Electric Power Hospital, 2-1-7 Fukushima, Fukushima-ku, Osaka-shi, Osaka 553-0003, Japan; ^2^Department of Physical Therapy, School of Rehabilitation, Hyogo University of Health Sciences, 1-3-6 Minatojima, Chuou-ku, Kobe-shi, Hyogo 650-8530, Japan

## Abstract

**Background:**

The stair-climbing test was used to assess the exercise capacity before lung resection in subjects with lung cancer. However, few studies have systematically evaluated the role of this exercise methodology as a postoperative test. The aim of the present study was to assess whether the stair-climbing test findings reflect the postoperative decrease in pulmonary function.

**Methods:**

Twenty subjects with non-small-cell lung cancer who underwent lung resection were enrolled in the study. Perioperative functional evaluation comprised the pulmonary function test, stair-climbing test, and 6-min walk distance test (6MWD). A correlation analysis was performed between the postoperative percentages of pulmonary function with respect to preoperative values and the exercise capacity.

**Results:**

No correlation was noted between the percentage changes in pulmonary function and those in 6MWD. However, there was a significant correlation between the percentage changes in forced expiratory volume in 1 s and those in the altitude reached in the stair-climbing test (*r*=0.46, *p* < 0.05) and between the percentage changes in carbon monoxide lung diffusion capacity and those in the altitude (*r*=0.54, *p* < 0.05).

**Conclusions:**

The stair-climbing test findings might be effective at detecting changes in exercise capacity induced by postoperative decrease in pulmonary function.

## 1. Introduction

In subjects with lung cancer, lung resection causes the lung volume to decrease, leading to a reduction in the ventilation volume and size of the pulmonary vascular bed [1]. These changes disturb pulmonary function. Two studies [1, 2] have reported that percent vital capacity (%VC), percent forced expiratory volume in 1 s (%FEV_1.0_), and percent carbon monoxide lung diffusion capacity (%D_LCO_) decrease by approximately 20%–30% at 1 month after surgery. These changes in pulmonary function can lead to a postoperative reduction in exercise capacity. Recently, various reports [3–6] have demonstrated that exercise training after resection confers an increase in exercise capacity in this population. In these reports, exercise capacity was evaluated using the 6-min walk test [7]. However, Nomori et al. [8] reported no significant correlations between the postoperative decrease in pulmonary function and that in 6-min walk distance (6MWD). Thus, discrepant findings have been published regarding the association between 6MWD findings and lung resection outcomes.

According to the recommendation of the European Respiratory Society and the clinical guidelines of European Society of Thoracic Surgery [9], the stair-climbing test should be used as a first-line screening test to optimize perioperative management. The stair-climbing test has been conventionally used by thoracic surgeons to select patients prior to surgery [10, 11], and Bolton et al. [10] reported a strong relationship between the altitude reached in the stair-climbing test and pulmonary function before lung resection. In our previous study [12], the stair-climbing test results showed a significant deterioration at one month after lung resection; however, a significant change in the 6MWD was not observed. Therefore, the stair-climbing test, as compared to the 6MWD, might be more sensitive at detecting changes in cardiorespiratory fitness induced by lung resection. However, few studies have reported the relationship between the altitudes reached in the stair-climbing test and pulmonary function after lung resection. The aim of the present study was to assess whether the stair-climbing test findings adequately reflect the postoperative decrease in pulmonary function compared with the 6MWD findings.

## 2. Methods

### 2.1. Subjects

Twenty-three subjects with non-small cell lung cancer who underwent lung resection at our hospital from January to October 2014 were enrolled in the study after obtaining their informed consent. Three subjects were excluded from the study due to postoperative cardiopulmonary complications. The following complications were considered [11]: respiratory failure requiring mechanical ventilation for >48 h, pneumonia, atelectasis requiring bronchoscopy, pulmonary edema, pulmonary embolism, myocardial infarction, hemodynamically unstable arrhythmia requiring medical treatment, cardiac failure, and death. The remaining 20 subjects (12 men and 8 women) formed the database for analysis. This study protocol was approved by the Ethics Committee of the Kansai Electric Power Hospital (#2639).

### 2.2. Experimental Design

Perioperative functional evaluation comprised the pulmonary function test, stair-climbing test, and 6MWD. The pulmonary function test was conducted before and at 1 month after surgery. In the present study, the following parameters were considered: %VC, %FEV_1.0_, and %D_LCO_; these data were expressed as a percentage of the predicted values for the given age, sex, and height. The percentage changes in postoperative data from the preoperative values were also assessed.

### 2.3. Stair-Climbing Test

The stair-climbing test was performed before, at 1 week after, and at 1 month after surgery. Our hospital has 35 flights of stairs, with each flight comprising 20–31 steps. Each step is 0.18-0.19 m in height. The subjects were asked to climb, at a pace of their own choice, the maximum number of steps possible and to stop only due to exhaustion, limiting dyspnoea, leg fatigue, or chest pain [13]. The subjects were accompanied by a physical therapist during the exercise who encouraged them to continue the test. During the test, pulse rate and capillary oxygen saturation were monitored using a portable pulse oximeter. For each subject, the number of steps climbed was recorded, and the altitude reached in the test was calculated as follows: height of the step in meters × steps climbed [14]. The percentage changes in postoperative data from the preoperative values were also assessed.

### 2.4. Six-Minute Walk Distance

The 6MWD was measured on the same day as the stair-climbing test, and the ATS reference guideline was followed [7]. The subjects were instructed to cover the greatest distance possible during the allotted time, at a self-determined walking speed, pausing to rest as needed. Every 1 min, the subjects were encouraged using standardized phrases. The 6MWD was measured before the stair-climbing test. The percentage changes in postoperative data from the preoperative values were also assessed.

### 2.5. Statistical Analysis

Data are expressed as mean ± standard deviation. The paired Student's *t*-test was used to determine the significance of changes in pulmonary function measured before and after surgery. Preoperative and repeat postoperative (at 1 week and 1 month) values of the altitude and 6MWD were compared using the repeated measures analysis of variance, with adjusted (Bonferroni *post hoc* test) pairwise comparisons. A correlation analysis between the postoperative percentages at 1 month of pulmonary function with respect to preoperative values and the altitude or 6MWD was performed using the Spearman's rank correlation test. Differences with *p* values of <0.05 were considered significant.

## 3. Results

Table 1 shows the characteristics of the subjects analyzed in this study. Fifteen subjects underwent pulmonary lobectomy, and five underwent wedge/segmentectomy. The results of the pulmonary function test, stair-climbing test, and 6MWD after resection are shown in Table 2. At 1 month after surgery, the %VC, %FEV_1.0_, and %D_LCO_ values were 79.5%, 82.9%, and 72.6% of the preoperative values, respectively.

**Figure 1:**
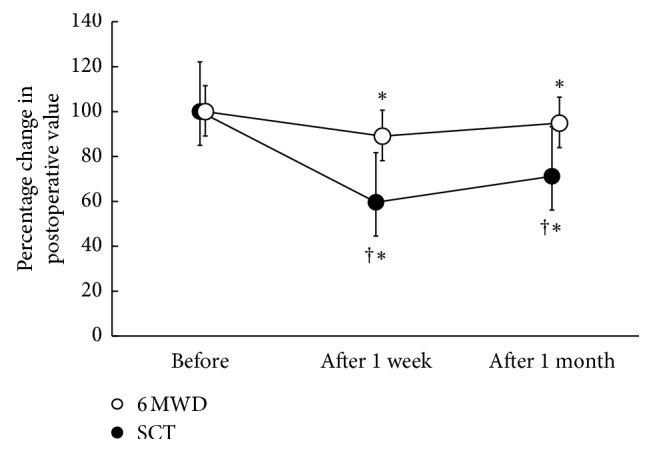
Percentage changes in postoperative SCT and 6MWD values compared with preoperative values. SCT, stair-climbing test; 6MWD, 6-min walk distance. Values are expressed as mean ± standard error. ^*∗*^*p* < 0.05: compared with preoperative values. ^†^*p* < 0.05: SCT versus 6MWD.

**Figure 2:**
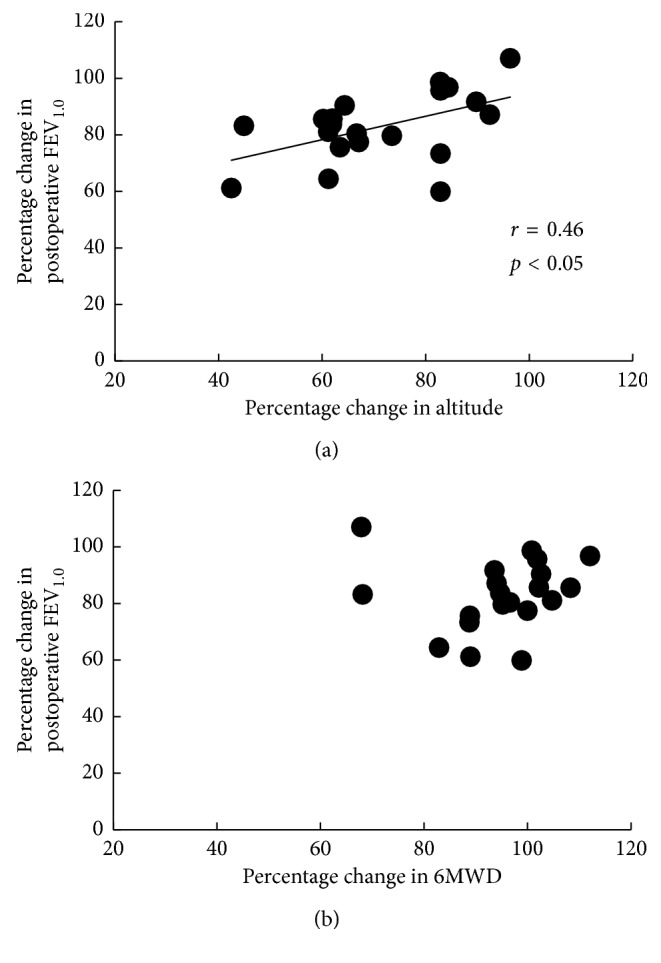
Correlation between percentage changes in altitude reached in the (a) stair-climbing test and (b) 6MWD and those in FEV_1.0_ values at 1 month after surgery. Altitude, altitude reached in the stair-climbing test; 6MWD, 6-min walk distance; FEV_1.0_, forced expiratory volume in 1 s.

**Figure 3:**
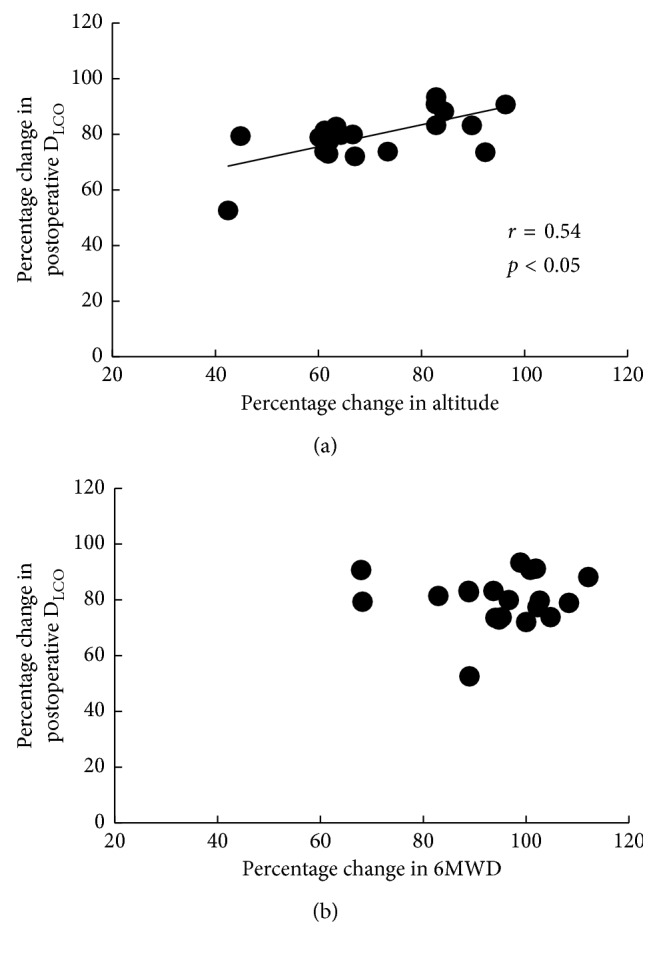
Correlation between percentage changes in altitude reached in the (a) stair-climbing test and (b) 6MWD and those in D_LCO_ values at 1 month after surgery. Altitude, altitude reached in the stair-climbing test; 6MWD, 6-min walk distance; D_LCO_, carbon monoxide lung diffusion capacity.

The stair-climbing and 6-min walk tests were tolerated by all subjects; moreover, no individual complained of angina, dizziness, or palpitations nor did anyone fall during the tests. Compared with the 6MWD, the percentage changes in postoperative altitude reached in the stair-climbing test from the preoperative values were significantly lower at 1 week and 1 month after resection (Figure 1). No correlation was noted between the percentage changes in pulmonary function and the percentage changes in 6MWD. However, there was a significant correlation between the changes in %FEV_1.0_ and the percentage changes in the altitude reached in the stair-climbing test (*r*=0.46, *p*=0.043; Figure 2) and between the changes in %D_LCO_ and the percentage changes in the altitude (*r*=0.54, *p*=0.01; Figure 3).

## 4. Discussion

The major finding of our study is that there was a significant correlation between the percentage changes in pulmonary function and those in the altitude reached in the stair-climbing test.

The reductions in ventilatory capacity, diffusing capacity, and maximal cardiac output after lung resection have been shown to be limiting factors for the reduction in exercise capacity [15]. In the present study, the percentage changes in 6MWD recovered up to approximately 90% from 1 week to 1 month after lung resection, as described in previous reports [4, 16]. By contrast, the percentage changes in the altitude reached in the stair-climbing test only recovered to 71.1% at 1 month after lung resection. Both the ventilation and oxygen consumption achieved in the stair-climbing test were significantly greater than those achieved in the walking test [17, 18]. Furthermore, in our previous study [12], both the preoperative and postoperative heart rates were significantly higher after the stair-climbing test than after the 6-min walk test. Thus, exercise intensity in the stair-climbing test could be higher than that in the 6-min walk test, which might lead to differences in the percentage changes in both the low-technology exercise tests after lung resection. Accordingly, the percentage changes in the altitude reached in the stair-climbing test were significantly lower than those in the 6MWD.

In our study, there were significant correlations between the changes in %FEV_1.0_ and %D_LCO_ and the percentage changes in the altitude reached in the stair-climbing test. Polletier et al. [19] reported that there was a significant correlation between the changes in %FEV_1.0_ and the exercise capacity after lung resection. Furthermore, D_LCO_ is related to the maximal oxygen uptake in humans [20]. Therefore, a reduction in pulmonary function might lead to a reduction in stair-climbing test findings. By contrast, no correlation was noted between the percentage changes in pulmonary function and those in 6MWD. Nomori et al. [8] reported that there was no significant correlation between the percentage decrease in 6MWD at 2 weeks after surgery and the percentage changes in pulmonary function. Roul et al. [21] suggested that in cases of 6MWD < 300 m, there was a significant correlation between the 6MWD and the ${\dot{\text{V}}\text{O}}_{2}$ peak. In our study, the mean 6MWD after lung resection was >300 m. Thus, following lung resection, the 6-min walk test is likely to measure patients' daily activity levels rather than the true maximal capacity. This could explain why the 6-min walk test might not be sensitive for detecting changes in cardiopulmonary fitness induced by lung resection compared with the stair-climbing test.

## 5. Conclusion

There was a significant correlation between the percentage changes in pulmonary function and those in the altitude reached in the stair-climbing test; however, a significant correlation in the 6MWD was not observed. Therefore, the present study demonstrated that the stair-climbing test findings might be effective at detecting changes in cardiorespiratory fitness induced by postoperative decrease in pulmonary function.

## Figures and Tables

**Table 1:** Characteristics of the subjects in the study.

	Mean ± SD	Range
Age (years)	67.1 ± 10.4	43–83
BMI (kg/m^2^)	22.4 ± 2.8	16.9–27.4
Hospital length of stay (days)	17.6 ± 5.7	11–31
Preoperative %VC	100.2 ± 15.0	84.0–136.9
Preoperative % FEV_1.0_	97.2 ± 19.9	56.8–133.2
Preoperative %D_LCO_	22.5 ± 12.8	65.7–166.9
Preoperative altitude (m)	22.5 ± 12.8	6.66–48.66
Preoperative 6MWD (m)	481.8 ± 107.3	265–630

%VC, % vital capacity; %FEV_1.0_, % forced expiratory volume in 1 s; %D_LCO_, % carbon monoxide lung diffusion capacity; altitude, altitude reached in the stair-climbing test; 6MWD, 6-min walk distance.

**Table 2:** Results of pulmonary function and exercise capacity after lung resection.

	1 week	1 month
Postoperative %VC	—	79.6 ± 15.5
Postoperative %FEV_1.0_	—	80.2 ± 18.7
Postoperative %D_LCO_	—	77.4 ± 23.3
Postoperative altitude (m)	13.8 ± 10.7	16.0 ± 9.9
Postoperative 6MWD (m)	427.1 ± 113.0	458.3 ± 118.5

%VC, % vital capacity; %FEV_1.0_, % forced expiratory volume in 1 s; %D_LCO_, % carbon monoxide lung diffusion capacity; altitude, altitude reached in the stair-climbing test; 6MWD, 6-min walk distance.

## Data Availability

The data used to support the findings of this study are included within the article.
